# Massive Malignant Epithelioid Angiomyolipoma of the Kidney

**DOI:** 10.15586/jkcvhl.v9i2.210

**Published:** 2022-04-22

**Authors:** Isaac M. Tessone, Benjamin Lichtbroun, Arnav Srivastava, Alexandra L. Tabakin, Charles F. Polotti, Roman Groisberg, Evita Sadimin, Eric A. Singer, Miral S. Grandhi

**Affiliations:** 1 Section of Urologic Oncology, Rutgers Cancer Institute of New Jersey and Rutgers Robert Wood Johnson Medical School, New Brunswick, NJ, USA;; 2 Division of Medical Oncology, Rutgers Cancer Institute of New Jersey and Rutgers Robert Wood Johnson Medical School, New Brunswick, NJ, USA;; 3 Section of Urologic Pathology, Rutgers Cancer Institute of New Jersey and Rutgers Robert Wood Johnson Medical School, New Brunswick, NJ, USA;; 4 Division of Surgical Oncology, Rutgers Cancer Institute of New Jersey and Rutgers Robert Wood Johnson Medical School, New Brunswick, NJ, USA

**Keywords:** epithelioid angiomyolipoma, mTOR inhibitor, PEComa, renal tumor, tuberous sclerosis

## Abstract

Renal angiomyolipomas (AMLs) are a subset of perivascular epithelioid cell neoplasms (PEComas) that are associated with tuberous sclerosis complex (TSC). Epithelioid angiomyolipomas (EAMLs) are a rare variant of AML with more aggressive propensities. EAMLs with malignant potential can be difficult to distinguish from relatively benign AMLs and other renal tumors. Although there are no established criteria for predicting EAML malignancy, there are proposed histologic parameters that are associated with higher tumor risk. EAML can be treated with surgical resection as well as mTOR inhibitors. Here, we present a unique case of a patient with a 36-cm renal EAML metastatic to the lungs that was treated with complete surgical resection of the primary lesion and mTOR inhibition.

## Introduction

Perivascular epithelioid cell neoplasms (PEComas) are a family of tumors originating from the mesenchymal tissue. They are characterized by histological evidence of melanocytic and smooth muscle markers (1). Renal angiomyolipomas (AMLs), a subset of PEComas, are kidney tumors composed of smooth muscle, mature adipose tissue, and thick-walled blood vessels. Renal AMLs typically exhibit benign tumor characteristics and are frequently associated with tuberous sclerosis complex (TSC) (2). Epithelioid angiomyolipomas (EAMLs) are a rare variant of AML with the potential for aggressive biological behavior, with approximately 20% of patients presenting with local invasion or metastasis (3). Appropriately distinguishing between classic benign AMLs and EAMLs with malignant potential is critically important for treatment and prognosis. Here, we present the case of a patient with a massive metastatic renal EAML.

## Case Report

**Figure 1:**
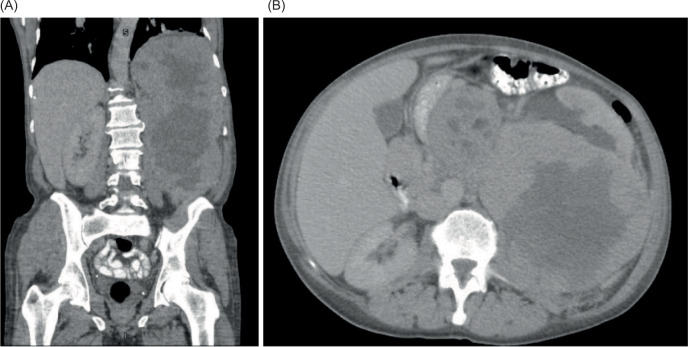
Abdominal CT images with contrast. CT images of the abdomen in coronal (A) and axial (B) planes with contrast enhancement demonstrating a large left retroperitoneal mass.

A 57-year-old man presented to his pulmonologist with worsening headaches and sinus congestion. This ultimately prompted computed tomography (CT) imaging which revealed a small left lung effusion, two nodules of the right lung, left hemidiaphragmatic elevation, and a 20-cm centrally necrotic neoplasm in the left upper quadrant (Figure 1). Positron emission tomography (PET) re-demonstrated the retroperitoneal mass, measuring 30 × 16 × 11 cm with a standardized uptake value (SUV) of 7.0, suggestive of malignancy. One fine-needle aspiration (FNA) sample and four core needle biopsy samples were obtained, revealing a malignant PEComa. Magnetic Resonance Imaging (MRI) demonstrated no brain metastases. However, further axial imaging revealed pelvic adenopathy and multiple pulmonary nodules, indicative of metastatic disease.

**Figure 2:**
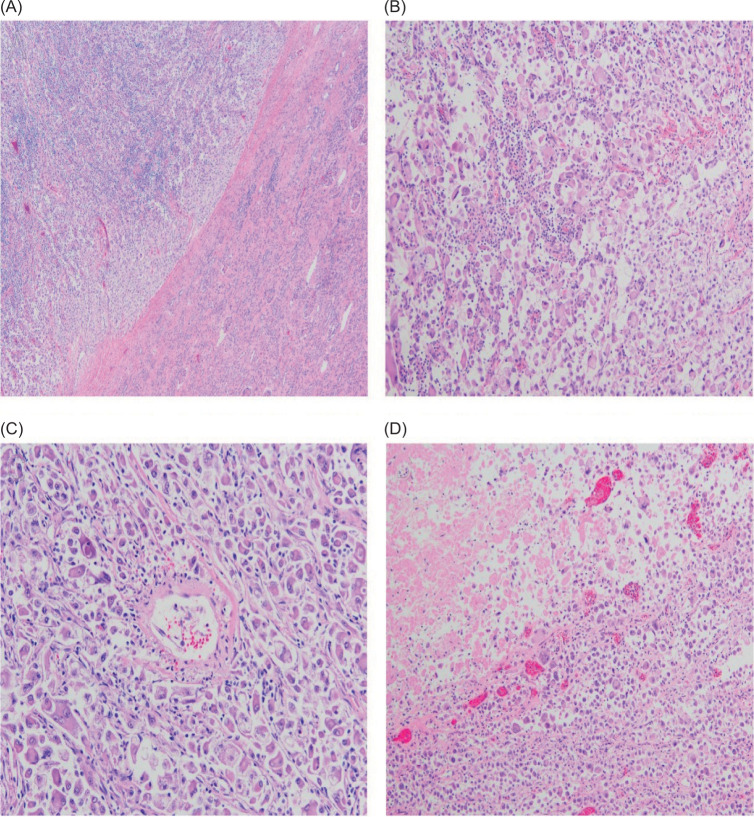
Primary tumor histology. Under low magnification (×40), the lesion can be seen arising in the kidney (A). Under higher magnification (×100), the lesion is composed of pleomorphic eosinophilic cells with prominent nucleoli (B), with some tumor cells intimately associated with vessels (C), and extensive necrosis (D).

In an operation intervention utilizing a multidisciplinary approach, the left retroperitoneal tumor was excised en bloc- with left nephrectomy, left adrenalectomy, and regional lymphadenectomy. Final pathology revealed a 36-cm EAML with extension into the adrenal gland and perinephric tissue (Tumor stage: T4N0M1). On microscopy, atypical mitoses, pleomorphic eosinophilic cells with prominent nucleoli, and extensive necrosis were identified (Figure 2). Positive margins were present at the perivascular and periureteral margins of resection, while all other margins were negative for tumor. In addition, genetic analysis of the tumor resection demonstrated a TSC2 pathogenic variant.

**Figure 3:**
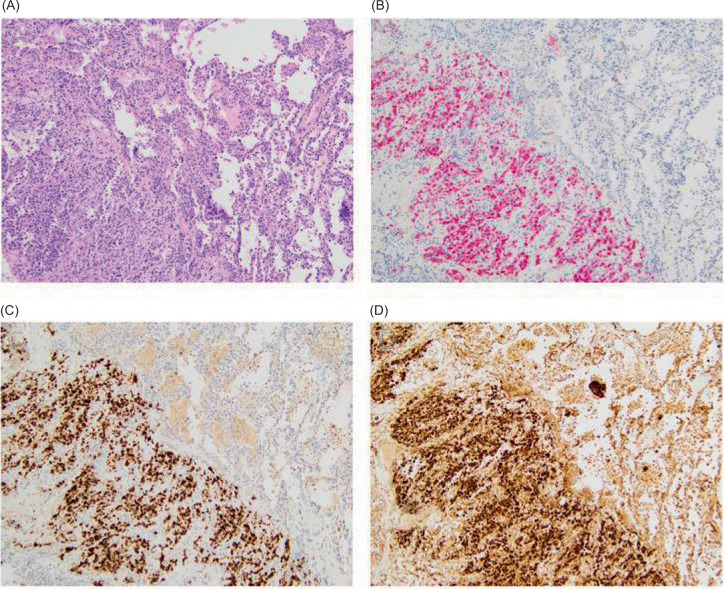
Lung metastasis tumor histology with immunohistochemical staining. The metastatic foci in the lung (original magnification ×100) have similar morphology compared to the primary lesion on H&E (A). By immunohistochemistry, these lesions are positive for MART1 (B), HMB45 (C), and Cathepsin K (D).

Two months postoperatively, a chest CT revealed new pulmonary nodules as well as the growth of his dominant lung nodule. The patient underwent video-assisted thoracoscopic surgery (VATS) with wedge resection of nodules in the right upper and lower lobes for the purpose of pathologic diagnosis of these lung nodules. Final pathology demonstrated metastatic EAML, measuring 2.7 cm in the right lower lobe and 1.1 cm in the right upper lobe. Surgical margins were negative. On immunohistochemistry, both the primary lesion and the metastatic foci were positive for MART1, HMB45, and Cathepsin K, patchy positive for SMA, and negative for Pankeratin, PAX8, S100, and Inhibin (Figure 3).

**Figure 4:**
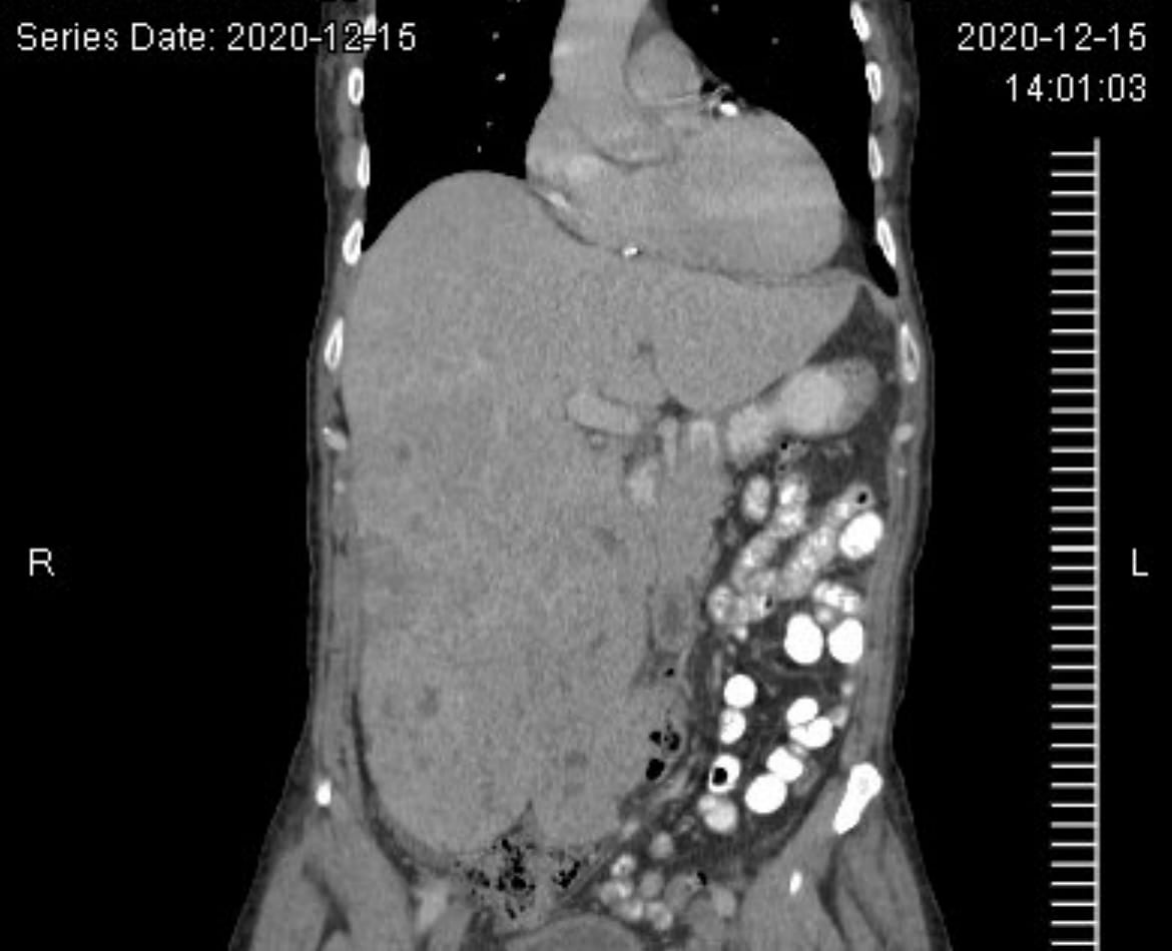
Abdominal CT image. CT image demonstrating massive tumor recurrence in the liver after surgical resection of primary tumor and prior to initiation of temsirolimus.

At 4 months follow-up from the VATS, a restaging CT scan demonstrated an enlarged liver with numerous low-density masses within the liver, including a conglomerate of masses within the right lobe of the liver, and an increase in the size and number of pulmonary nodules (Figure 4). The restaging CT also demonstrated the development of ascites in the pelvis and a probable tumor implant in the left peritoneal cavity, lateral to the psoas muscle. The patient was started on systemic temsirolimus, an mTOR inhibitor. Initially, after 3 months on temsirolimus, the patient demonstrated a dramatic improvement both clinically and radiographically. However, he ultimately stopped responding to temsirolimus. He was then transitioned to gemcitabine but unfortunately, the patient eventually passed away after this treatment alteration.

## Discussion

The diagnosis of a renal EAML can be challenging and elusive as there are no pathognomonic clinical manifestations or imaging characteristics. Specifically, EAMLs are often difficult to distinguish radiographically and histologically from other tumors such as renal cell carcinomas (RCCs), leading to potential misdiagnoses (4, 5). Nonetheless, indicators of a potential EAML diagnosis can be gleaned from radiologic findings. In a study by Zhong et al., renal EAMLs displayed certain characteristics on MRI such as large size (mean diameter of 7.1 cm), exophytic growth, minimal macroscopic fat, microscopic fat, enlarged vessels, massive hemorrhage, and hypointensity on T2 weighted imaging (6). Furthermore, another study of nine EAML cases indicated that the radiologic finding of a lipid-poor mass without calcification should similarly raise clinical suspicion for a renal EAML diagnosis (7).

Beyond imaging characteristics, a retrospective analysis comparing patients with classic AML (n = 204) and EAML (n = 27) demonstrated that younger age, male sex, and larger tumor size were predictive of EAML (8). Even so, a definitive diagnosis is based on histological analysis of the tumor. While by immunohistochemistry AML and EAML express a similar profile, morphologically the mesenchymal component of EAML shows predominantly large eosinophilic pleomorphic cells with prominent nucleoli, compared to the bland spindled cells seen in classic AML. In addition, the presence of atypical mitoses and necrosis also supports an EAML diagnosis, as these features should not be present in classic AML. Furthermore, the positive expression for HMB-45 along with a lack of expression of cytokeratins and S100 rules out RCC and melanoma, which are also in the differential diagnosis.

While classic renal AMLs are generally viewed as benign, EAMLs can often undergo malignant transformation, as demonstrated in this report. In one study examining a cohort of 41 patients with EAML, 48.5% of patients developed metastases and 33% had died due to the disease at a mean follow-up of 44.5 months (9).

Nevertheless, due to the rarity of EAMLs, no established criteria exist for predicting malignancy. In a 2010 study, Brimo et al. proposed that four specific histologic features are predictive of malignant behavior when at least three are present (10). These features are ≥70% atypical epithelioid cells, ≥two mitotic figures per 10 hpf, atypical mitotic figures, and necrosis. Indeed, the pathology of the tumor in this report meets three of these parameters as it demonstrated 12 mitotic figures per 10 hpf, the presence of atypical mitoses, and necrosis. Nese et al. similarly proposed five parameters for stratifying EAMLs according to the risk of malignant progression, with <two parameters considered low risk for progression, two or three parameters considered intermediate risk, and >three parameters considered high risk. The parameters included TSC or concurrent AML, necrosis, tumor size >7 cm, extrarenal invasion and/or renal vein involvement, and carcinoma-like growth pattern (9). The tumor in this case demonstrated necrosis, size >7 cm, and extrarenal invasion, consistent with an intermediate risk of progression.

Complete surgical resection remains the preferred treatment option for many kidney cancers such as RCCs and PEComas (11, 12). However, a substantial and growing body of evidence suggests utility in the systemic treatment of metastatic PEComas with mTOR inhibitors (13–16). Most notably, a recent clinical trial on treating PEComas with nab-sirolimus, an albumin-bound mTOR inhibitor, demonstrated favorable and durable responses, and subsequently obtained FDA approval for the treatment of locally advanced unresectable or metastatic PEComa (17).

Alterations in the mTOR pathway are thought to underlie the development of AMLs. Specifically, mutations in TSC1 and TSC2 promote the disinhibition of the mTOR pathway. This leads to dysregulation of the cell cycle and an increase in vascular endothelial growth factor (VEGF) expression, contributing to tumor proliferation and vascularization (15, 18). In this case report, the tumor demonstrated a TSC2 mutation and the patient was started on temsirolimus, an mTOR inhibitor. Furthermore, there are genetic alterations of the mTOR pathway, aside from TSC1 and TSC2 mutations, that also drive PEComa growth. These other genetic alterations can point the way to future therapeutic targets (19).

There are currently several other ongoing trials focused on PEComa treatments. One trial is investigating the combination of nivolumab with escalating doses of nab-sirolimus in treating PEComas (20). Another trial by the National Cancer Institute (NCI) is investigating the response of PEComas and other rare tumors to treatment with nivolumab alone versus nivolumab and ipilimumab (21). Another study out of France is investigating the potential role of beta blocker therapy in treating PEComas after noting some similarities between PEComas and infantile hemangiomas, which can be effectively treated with propanol (22).

## Conclusion

While often thought of as benign, some renal AMLs—specifically EAMLs—may exhibit aggressive features, including distant metastases. As our understanding of renal EAML grows, it remains crucial to promptly diagnose and treat these potentially malignant tumors. Complete surgical resection remains the mainstay of treatment, yet some patients may benefit from systemic therapy, particularly with mTOR inhibition. As with many rare tumors, a multidisciplinary approach at high volume centers should be considered.
